# Supplementary material for: Characteristic Gut Bacteria in High Barley Consuming Japanese Individuals without Hypertension
Source: Microorganisms. 2023 May 9;11(5):1246. doi: 10.3390/microorganisms11051246 (PMC10223911; doi:10.3390/microorganisms11051246)
Supplement: Supplementary file 1 [file microorganisms-11-01246-s001.zip › Supplementary Figure.pdf]

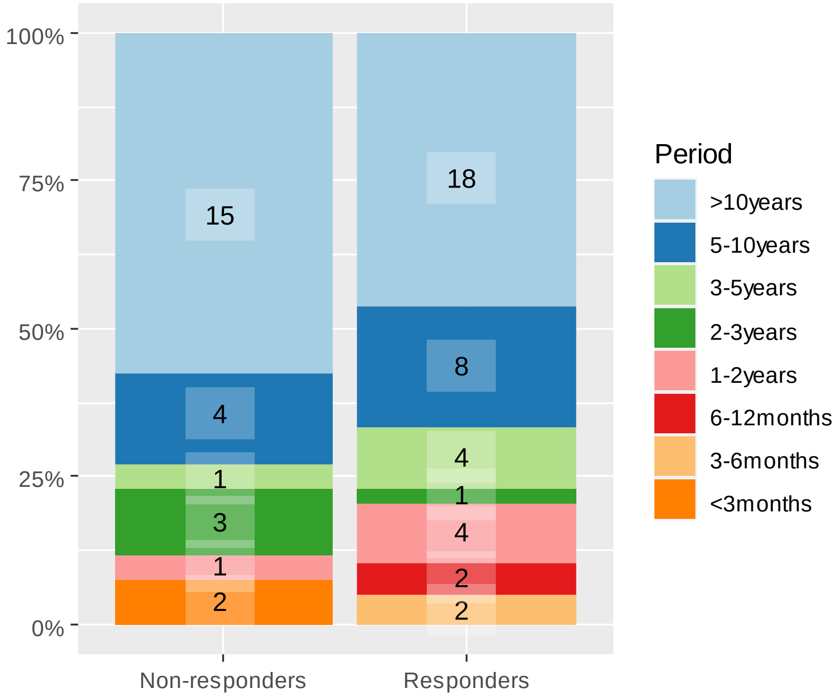

Figure S1: Distribution of barley intake duration in responders and non-responders. (*p* = 0.92, Fisher's exact test).

(A)

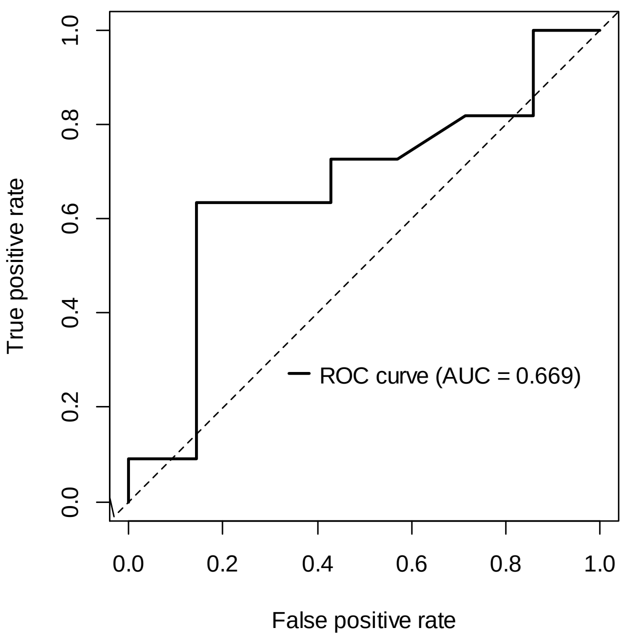

(B)

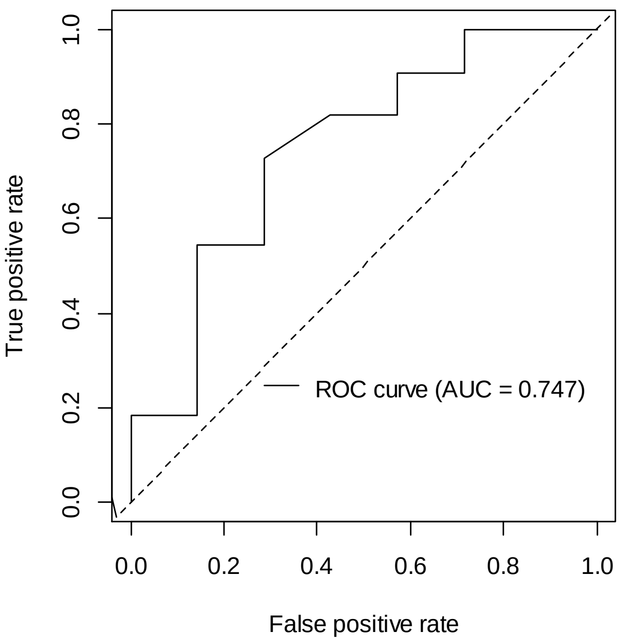

(C)

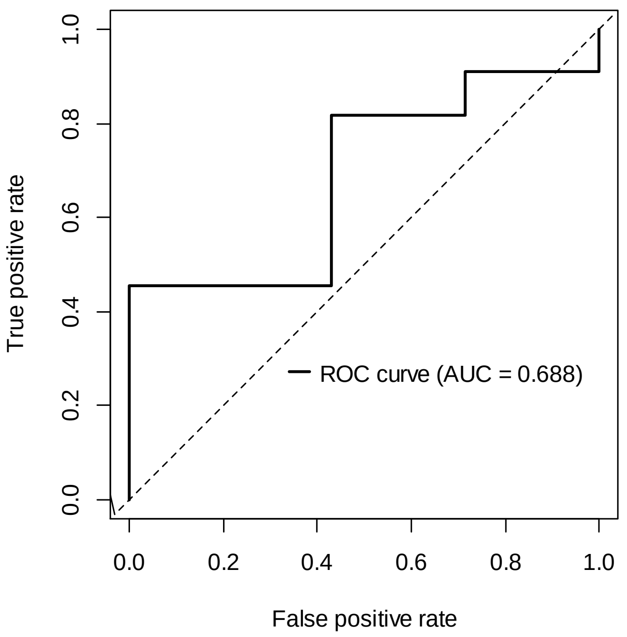

Figure S2: Representative ROC curves for different gut bacteria included as explanatory variables.
(A) ROC curves for the models that were the most accurate when built based on the 50 genera of gut bacteria. The optimal cutoff for this model was 0.488, with a sensitivity of 0.64, specificity of 0.86, and overall accuracy of 0.75.
(B) ROC curves for the models that were the most accurate when built based on 20 genera of gut bacteria. The optimal cutoff for this model was 0.520, with a sensitivity of 0.64, specificity of 0.86, and overall accuracy of 0.75.
(C) ROC curves for the models that were the most accurate when built based on 100 genera of gut bacteria. The optimal cutoff for this model was 0.682, with a sensitivity of 0.46, specificity of 1.0, and overall accuracy of 0.73.
