# Supplementary material for: Characteristic Gut Bacteria in High Barley Consuming Japanese Individuals without Hypertension
Source: Microorganisms. 2023 May 9;11(5):1246. doi: 10.3390/microorganisms11051246 (PMC10223911; doi:10.3390/microorganisms11051246)
Supplement: Supplementary file 1 [file microorganisms-11-01246-s001.zip › Supplementary Table.pdf]

Table S1 Characteristics of medical checkup and dietary habits in high and low risk hypertension groups in the low barley group.

	High risk (n = 28)	Low risk (n = 37)	p value [(1)]
	n (%) or mean (SD)	n (%) or mean (SD)	
Male (n)	24 (86%)	28 (77%)	0.49 [(2)]
Age (years)	52.4 (6.2)	49.9 (6.0)	0.11
Barley intake (g/1000 kcal)	1.5 (1.2)	1.5 (1.3)	0.88
Medications of hypertension drug (n)	10 (36%)	0 (0%)	<0.001 [(2)]
Parents with hypertension (n)	17 (61%)	16(43%)	0.25
Medical checkup			
Weight (kg)	71.0 (12.9)	62.4 (10.7)	0.005
BMI (kg/m^2)	24.4 (4.0)	22.0 (3.3)	0.01
SBP (mmHg)	134 (8)	112 (10)	<0.001
DBP (mmHg)	86 (6)	71 (8)	<0.001
Fasting blood glucose (mg/dL)	97 (10)	95 (16)	0.64
HbA1c (%)	5.6 (0.3)	5.5 (0.4)	0.52
Triglycerides (mg/dL)	124 (53)	126 (105)	0.94
HDL-cholesterol (mg/dL)	62 (12)	60 (17)	0.68
LDL-cholesterol (mg/dL)	130 (31)	118 (26)	0.09
Nutrients			
Energy (kcal/d)	1735 (382)	1763 (561)	0.82
Protein (g/d)	59 (18)	58 (18)	0.79
Fat (g/d)	49 (16)	48 (16)	0.89
Carbohydrate (g/d)	210 (61)	215 (68)	0.75
Sodium chloride (g/d)	10.5 (2.3)	10.2 (3.3)	0.67
Lifestyle			
Smoking (present, past, never) (n)	9, 9, 10 (32%, 32%, 36%)	17, 9, 11 (46%, 24%, 30%)	0.53 [(2)]
With physical activity [(3)] (n)	9 (32%)	14 (38%)	0.83 [(2)]

[(1)] High risk group and low risk group were compared using Student's *t* -test.

[(2)] High risk group and low risk group were compared using Pearson’s chi-squared test.

[(3)] Light exercise for a total of at least 30 min/day, at least twice a week, for at least 1 year.

Table S2 Measurements of the 50 dominant genera in all participants, responders, non-responders, low risk group, and high risk group subjects.

	All participants (n = 130)				High barley group							Low barley group						
					responders (n = 39)			non-responders (n = 26)				Low risk (n = 37)			High risk (n = 28)			
	Mean	Median	1st Qu	3rd Qu	Median	1st Qu	3rd Qu	Median	1st Qu	3rd Qu	*p* -value	Median	1st Qu	3rd Qu	Median	1st Qu	3rd Qu	*p* -value
Bacteroides	27.36	26.43	14.92	36.95	26.05	16.07	34.75	27.27	16.00	36.66	0.98	22.95	13.56	35.98	35.09	17.44	41.72	0.10
Prevotella 9	7.07	0.01	0.00	0.03	0.00	0.00	0.01	0.01	0.00	24.19	0.03	0.01	0.00	14.37	0.01	0.00	0.01	0.16
Blautia	6.06	5.15	2.79	8.36	5.48	4.01	8.95	3.66	1.96	8.94	0.12	4.71	2.55	5.71	5.78	3.10	9.78	0.15
Bifidobacterium	6.00	2.94	1.09	7.54	5.02	2.33	11.40	3.21	0.82	6.37	0.09	2.28	1.31	5.83	2.23	0.42	4.62	0.47
Faecalibacterium	4.90	3.36	0.65	7.73	5.86	2.09	9.28	1.74	0.54	5.28	0.02	3.19	1.17	8.21	2.75	0.33	4.57	0.40
Megamonas	3.28	0.00	0.00	3.60	0.00	0.00	0.01	0.00	0.00	4.44	0.09	0.00	0.00	2.05	0.19	0.00	6.82	0.31
Parabacteroides	2.55	1.84	0.82	3.22	1.30	0.53	2.52	1.35	0.91	2.19	0.46	2.28	1.11	4.46	2.22	1.39	3.56	0.99
Sutterella	1.86	1.59	0.42	2.67	1.35	0.34	2.34	1.95	1.05	2.88	0.23	1.25	0.37	2.27	1.62	0.64	3.43	0.11
Lachnoclostridium	1.85	1.35	0.73	2.29	1.13	0.71	1.75	2.12	0.93	2.84	0.02	1.12	0.72	2.12	1.72	0.84	2.96	0.11
Ruminococcus torques group	1.83	1.22	0.62	2.29	1.06	0.55	1.86	1.46	0.50	2.76	0.36	1.47	0.72	2.58	1.21	0.73	2.04	0.47
Collinsella	1.81	1.33	0.48	2.23	1.00	0.11	2.71	1.41	0.83	2.41	0.45	1.43	0.68	2.12	1.30	0.36	1.89	0.44
Eubacterium rectale group	1.67	1.00	0.07	2.46	1.58	0.01	3.11	0.40	0.03	2.27	0.29	0.85	0.37	1.90	1.06	0.10	2.73	0.70
Fusobacterium	1.62	0.00	0.00	0.72	0.00	0.00	0.43	0.16	0.00	1.02	0.08	0.00	0.00	0.01	0.06	0.00	0.90	0.10
Anaerostipes	1.44	0.89	0.18	2.02	1.53	0.46	3.97	0.99	0.30	1.84	0.095	0.49	0.12	1.47	0.30	0.09	1.88	0.75
Subdoligranulum	1.44	0.97	0.02	2.15	1.69	0.09	2.58	0.26	0.00	1.39	0.04	1.24	0.01	2.71	0.88	0.11	1.88	0.65
Fusicatenibacter	1.37	0.94	0.16	2.16	1.02	0.49	2.20	0.67	0.05	1.09	0.16	1.21	0.10	2.27	0.96	0.18	1.93	0.55
Alistipes	1.20	0.40	0.01	1.05	0.13	0.01	0.85	0.35	0.05	0.99	0.33	0.72	0.07	2.18	0.45	0.01	0.96	0.21
Phascolarctobacterium	1.15	0.55	0.00	2.10	0.17	0.00	1.75	0.62	0.00	2.22	0.19	1.20	0.00	2.55	1.12	0.02	2.20	0.98
Eubacterium hallii group	1.09	0.76	0.17	1.57	0.78	0.24	1.64	0.66	0.29	1.02	0.46	0.56	0.10	1.20	0.83	0.20	1.88	0.43
Megasphaera	1.04	0.00	0.00	1.30	0.00	0.00	0.55	0.00	0.00	1.42	0.54	0.00	0.00	1.50	0.00	0.00	0.97	0.35
Prevotella 2	0.95	0.00	0.00	0.00	0.00	0.00	0.00	0.00	0.00	0.00	0.94	0.00	0.00	0.00	0.00	0.00	0.00	0.37
Roseburia	0.91	0.57	0.13	1.13	0.62	0.10	1.08	0.34	0.13	0.86	0.47	0.67	0.24	1.53	0.31	0.13	0.93	0.23
Alloprevotella	0.86	0.00	0.00	0.00	0.00	0.00	0.00	0.00	0.00	0.00	0.86	0.00	0.00	0.00	0.00	0.00	0.00	0.75
Klebsiella	0.81	0.00	0.00	0.12	0.01	0.00	0.23	0.00	0.00	0.01	0.21	0.00	0.00	0.03	0.00	0.00	0.13	0.71
Lachnospira	0.78	0.36	0.02	1.05	0.62	0.22	1.37	0.15	0.00	0.52	0.02	0.36	0.04	0.90	0.51	0.00	1.05	0.99
Escherichia Shigella	0.77	0.06	0.01	0.26	0.06	0.01	0.30	0.10	0.03	0.31	0.28	0.05	0.01	0.20	0.05	0.00	0.20	0.60
Prevotellaceae NK3B31 group	0.73	0.00	0.00	0.00	0.00	0.00	0.00	0.00	0.00	0.00	0.36	0.00	0.00	0.00	0.00	0.00	0.00	0.80
Lachnospiraceae uncultured	0.66	0.44	0.17	0.87	0.44	0.17	0.93	0.38	0.15	0.79	0.61	0.48	0.18	0.76	0.42	0.25	1.00	0.81
Ruminococcus 2	0.66	0.00	0.00	1.01	0.00	0.00	0.94	0.00	0.00	0.42	0.80	0.00	0.00	1.05	0.00	0.00	1.39	0.63
Veillonella	0.63	0.02	0.00	0.21	0.06	0.01	0.34	0.01	0.00	0.07	0.10	0.01	0.00	0.04	0.03	0.00	0.41	0.24
Streptococcus	0.61	0.15	0.03	0.53	0.17	0.06	0.58	0.12	0.03	0.23	0.27	0.18	0.02	0.55	0.12	0.02	0.53	0.94
Akkermansia	0.54	0.00	0.00	0.02	0.00	0.00	0.00	0.00	0.00	0.01	0.27	0.00	0.00	0.30	0.00	0.00	0.03	0.73

Butyricicoccus	0.54	0.47 [0.27, 0.74]	0.61 [0.32, 0.80]	0.48 [0.30, 0.68]	0.45	0.36 [0.20, 0.61]	0.55 [0.30, 0.75]	0.15
Acidaminococcus	0.53	0.00 [0.00, 0.69]	0.00 [0.00, 0.88]	0.00 [0.00, 0.69]	0.86	0.07 [0.00, 0.82]	0.00 [0.00, 0.43]	0.24
Dorea	0.50	0.37 [0.18, 0.66]	0.38 [0.13, 0.62]	0.45 [0.22, 0.58]	0.54	0.27 [0.21, 0.70]	0.33 [0.07, 0.80]	0.60
Lachnospiraceae UCG-008	0.44	0.29 [0.08, 0.66]	0.37 [0.10, 0.69]	0.29 [0.06, 0.57]	0.51	0.24 [0.08, 0.55]	0.37 [0.09, 0.77]	0.70
Ruminococcaceae uncultured	0.42	0.16 [0.05, 0.56]	0.20 [0.04, 0.46]	0.11 [0.03, 0.35]	0.37	0.39 [0.10, 0.85]	0.12 [0.04, 0.49]	0.05
Rhodospirillaceae uncultured	0.41	0.00 [0.00, 0.00]	0.00 [0.00, 0.00]	0.00 [0.00, 0.00]	0.09	0.00 [0.00, 0.22]	0.00 [0.00, 0.00]	0.25
Paraprevotella	0.40	0.00 [0.00, 0.08]	0.00 [0.00, 0.00]	0.00 [0.00, 0.28]	0.13	0.00 [0.00, 0.17]	0.00 [0.00, 0.01]	0.87
Ruminococcus 1	0.40	0.00 [0.00, 0.19]	0.00 [0.00, 0.64]	0.00 [0.00, 0.01]	0.14	0.01 [0.00, 0.37]	0.00 [0.00, 0.01]	0.06
Parasutterella	0.37	0.02 [0.00, 0.19]	0.05 [0.00, 0.41]	0.04 [0.00, 0.25]	0.96	0.00 [0.00, 0.08]	0.01 [0.00, 0.10]	0.84
Barnesiella	0.34	0.04 [0.00, 0.41]	0.01 [0.00, 0.20]	0.01 [0.00, 0.29]	0.77	0.06 [0.00, 0.82]	0.08 [0.00, 0.64]	0.72
Dialister	0.28	0.00 [0.00, 0.26]	0.00 [0.00, 0.59]	0.00 [0.00, 0.64]	0.81	0.00 [0.00, 0.10]	0.00 [0.00, 0.00]	0.19
Ruminococcaceae UCG-002	0.28	0.00 [0.00, 0.12]	0.00 [0.00, 0.02]	0.00 [0.00, 0.18]	0.11	0.00 [0.00, 0.25]	0.00 [0.00, 0.09]	0.42
Mitsuokella	0.26	0.00 [0.00, 0.00]	0.00 [0.00, 0.00]	0.00 [0.00, 0.00]	0.81	0.00 [0.00, 0.00]	0.00 [0.00, 0.00]	0.04
Eubacterium coprostanoligenes group	0.25	0.00 [0.00, 0.27]	0.00 [0.00, 0.17]	0.01 [0.00, 0.24]	0.78	0.01 [0.00, 0.53]	0.00 [0.00, 0.15]	0.34
Ruminiclostridium 5	0.24	0.10 [0.05, 0.30]	0.09 [0.05, 0.36]	0.08 [0.02, 0.22]	0.40	0.16 [0.06, 0.40]	0.10 [0.05, 0.23]	0.14
Ruminococcaceae UCG-013	0.24	0.13 [0.03, 0.31]	0.21 [0.08, 0.50]	0.08 [0.00, 0.19]	0.03	0.09 [0.01, 0.27]	0.13 [0.09, 0.30]	0.31
Lactobacillus	0.23	0.00 [0.00, 0.00]	0.00 [0.00, 0.01]	0.00 [0.00, 0.00]	0.26	0.00 [0.00, 0.00]	0.00 [0.00, 0.00]	0.09
Ruminococcus gauvreauii group	0.21	0.01 [0.00, 0.23]	0.01 [0.00, 0.29]	0.01 [0.00, 0.16]	0.80	0.01 [0.00, 0.30]	0.01 [0.00, 0.11]	0.78

p-values are the results of between-group comparisons by Mann–Whitney's U-test.

Bacteria were defined as dominant genera by selecting the 50 genera with the highest mean relative abundance in all participants.

Table S3 Measurements of six genera specific to responder subjects in responders and non-responders excluding subjects taking medications for hypertension.

	Non-respondents excluding use of hypertension medications (n = 19)						Responders (n = 39)						
	Median	[	1st Qu	,	3rd Qu	]	Median	[	1st Qu	,	3rd Qu	]	*p* -value
Prevotella 9	0.02	[	0.00	,	33.10	]	0.00	[	0.00	,	0.01	]	0.01
Faecalibacterium	1.23	[	0.54	,	4.14	]	5.86	[	2.09	,	9.28	]	0.01
Lachnoclostridium	2.53	[	1.41	,	2.97	]	1.13	[	0.71	,	1.75	]	0.002
Subdoligranulum	0.22	[	0.01	,	1.36	]	1.69	[	0.09	,	2.58	]	0.03
Lachnospira	0.13	[	0.01	,	0.43	]	0.62	[	0.22	,	1.37	]	0.02
Ruminococcaceae UCG-013	0.08	[	0.01	,	0.15	]	0.21	[	0.08	,	0.50	]	0.02

p -values are the results of between-group comparisons by Mann–Whitney's U-test.

Table S4 Association between responder characteristic bacteria and hypertension, adjusted for age, sex, BMI, lifestyle-related diseases (fasting blood glucose, LDL-cholesterol), and history of hypertension in parents using logistic regression analysis.[(1)]

	Model 1				Model 2				Model 3			
Variable	R	SE	*p* value		R	SE	*p* value		R	SE	*p* value	
Faecalibacterium	0.21	0.11	0.06	#								
Lachnoclostridium					-0.16	0.15	0.31					
Ruminococcaceae UCG-013									4.71	2.28	0.04	*
Sex (0: Male, 1: Female)	-0.43	1.13	0.70		-0.35	1.07	0.75		0.48	1.25	0.70	
Age (years)	-0.17	0.08	0.03	*	-0.09	0.06	0.10		-0.15	0.07	0.03	*
BMI (kg/m2)	-0.13	0.14	0.34		-0.15	0.13	0.25		-0.20	0.15	0.17	
Fasting blood glocose (mg/dL)	-0.08	0.05	0.13		-0.09	0.05	0.088	#	-0.11	0.06	0.053	#
Triglycerides (mg/dL)	0.00	0.01	0.69		0.00	0.00	0.90		0.00	0.01	0.56	
LDL-cholesterol (mg/dL)	-0.02	0.02	0.19		-0.02	0.01	0.14		-0.03	0.02	0.12	
Parents with hypertension (0: Yes, 1: No)	-1.89	1.04	0.07	*	-2.24	0.81	<0.01	**	-2.57	0.91	<0.01	**

	Model 4				Model 5				Model 6			
Variable	R	SE	*p* value		R	SE	*p* value		R	SE	*p* value	
Lachnospira	0.27	0.37	0.46									
Prevotella 9					-0.04	0.03	0.21					
Subdoligranulum									0.40	0.30	0.17	
Sex (0: Male, 1: Female)	-0.59	1.10	0.59		-0.27	1.04	0.79		-0.46	1.08	0.67	
Age (years)	-0.09	0.06	0.09	#	-0.10	0.06	0.08	#	-0.10	0.06	0.07	#
BMI (kg/m2)	-0.16	0.13	0.23		-0.15	0.13	0.25		-0.13	0.13	0.32	
Fasting blood glocose (mg/dL)	-0.08	0.06	0.164		-0.09	0.05	0.0998	#	-0.10	0.05	0.07	#
Triglycerides (mg/dL)	0.00	0.00	0.82		0.00	0.01	0.64		0.00	0.01	0.83	
LDL-cholesterol (mg/dL)	-0.02	0.01	0.12		-0.02	0.01	0.12		-0.02	0.01	0.16	
Parents with hypertension (0: Yes, 1: No)	-2.27	0.81	<0.01	**	-2.16	0.82	0.01	**	-2.22	0.82	<0.01	**

[(1)]Objective variable is 1: Responder, 0: Non-responder. Variance inflation factor (VIF) was less than 2 for all.

*: $p < 0.05$, **: $p < 0.01$, #: $p < 0.1$

Table S5 Association between responder characteristic bacteria and hypertension, adjusted for age, sex, BMI, lifestyle-related diseases (fasting blood glucose, LDL-cholesterol), and lifestyles (smoking, physical activity) using logistic regression analysis.[1]

	Model 1				Model 2				Model 3			
Variable	R	SE	*p* value		R	SE	*p* value		R	SE	*p* value	
Faecalibacterium	0.27	0.11	0.015	*								
Lachnoclostridium					-0.30	0.15	0.042	*				
Ruminococcaceae UCG-013									3.96	1.91	0.038	*
Sex (0: Male, 1: Female)	-0.69	1.24	0.58		-0.53	1.15	0.64		0.23	1.22	0.85	
Age (years)	-0.25	0.08	< 0.01	**	-0.17	0.06	< 0.01	**	-0.18	0.07	< 0.01	**
BMI (kg/m2)	-0.07	0.13	0.62		-0.11	0.12	0.38		-0.07	0.12	0.54	
Fasting blood glucose (mg/dL)	-0.10	0.06	0.099	#	-0.11	0.06	0.058	#	-0.12	0.06	0.035	*
Triglycerides (mg/dL)	0.00	0.01	0.99		0.00	0.00	0.88		0.00	0.01	0.91	
LDL-cholesterol (mg/dL)	-0.01	0.01	0.35		-0.01	0.01	0.39		-0.02	0.01	0.20	
Smoking (0: present, 1: past, 2: never)	-0.83	0.55	0.13		-0.79	0.55	0.15		-0.74	0.52	0.16	
Physical activity habits (0: yes, 1: no) [2]	-1.89	1.04	0.07	#	-2.38	1.08	0.03	*	-1.71	1.01	0.09	#

	Model 4				Model 5				Model 6			
Variable	R	SE	*p* value		R	SE	*p* value		R	SE	*p* value	
Lachnospira	0.71	0.43	0.099	#								
Prevotella 9					-0.05	0.03	0.097	#				
Subdoligranulum									0.54	0.30	0.069	#
Sex (0: Male, 1: Female)	-1.28	1.22	0.29		-0.43	1.08	0.69		-0.65	1.11	0.56	
Age (years)	-0.17	0.06	< 0.01	**	-0.16	0.06	0.01	*	-0.17	0.06	< 0.01	**
BMI (kg/m2)	-0.10	0.12	0.41		-0.06	0.11	0.60		-0.04	0.12	0.74	
Fasting blood glucose (mg/dL)	-0.08	0.06	0.143		-0.11	0.05	0.052	#	-0.11	0.06	0.053	#
Triglycerides (mg/dL)	0.00	0.00	0.98		0.00	0.00	0.76		0.00	0.01	0.86	
LDL-cholesterol (mg/dL)	-0.02	0.01	0.20		-0.02	0.01	0.21		-0.01	0.01	0.37	
Smoking (0: present, 1: past, 2: never)	-0.97	0.59	0.10	#	-0.71	0.52	0.17		-0.67	0.53	0.21	
Physical activity habits (0: yes, 1: no) [2]	-2.26	1.08	0.04	*	-1.53	0.98	0.12		-1.95	1.01	0.05	#

[1]Objective variable is 1: Responder, 0: Non-responder. All variance inflation factor (VIF) was less than 2.

[2]Physical activity habits: Light sweaty activity for a total of at least 30 minutes per day, at least twice a week, for at least 1 year.

*: $p < 0.05$, **: $p < 0.01$, #: $p < 0.1$
